# N-(2-hydroxyphenyl)acetamide (NA-2) and Temozolomide synergistically induce apoptosis in human glioblastoma cell line U87

**DOI:** 10.1186/s12935-014-0133-5

**Published:** 2014-11-30

**Authors:** Farina Hanif, Kahkashan Perveen, Huma Jawed, Aqeel Ahmed, Saima M Malhi, Siddiqua Jamall, Shabana U Simjee

**Affiliations:** Dr. Panjwani Center for Molecular Medicine and Drug Research, International Center for Chemical and Biological Sciences, University of Karachi, Karachi, 75270 Pakistan; H.E.J. Research Institute of Chemistry, International Center for Chemical and Biological Sciences, University of Karachi, Karachi, 75270 Pakistan; Department of Biochemistry, University of Karachi, Karachi, 75270 Pakistan

**Keywords:** Glioblastoma, Apoptosis, Bax-Bcl-2 ratio, NSAIDs, Temozolomide

## Abstract

**Background:**

Despite the modern therapies available for treating glioblastoma multiforme (GBM), it is still a deadly disease. The development of new therapeutic strategies for the management of gliomas is therefore crucial. The present study is designed to analyze the therapeutic potentials of synthetic compound N-(2-hydroxyphenyl)acetamide (NA-2) in the treatment of GBM as a single agent or in combination with Temozolomide (TMZ) on glioblastoma cells.

**Methods:**

MTT and TUNEL assays were used to detect the growth inhibitory effect and apoptotic activity of NA-2 alone and in combination with TMZ. Synergy was assessed using combination Index method. The expression of apoptosis related markers Bax, Bcl-2 and caspase-3 were assessed by RT-PCR, whereas, the active caspase-3 protein expression was determined using imunocytochemistry.

**Results:**

Both NA-2 and TMZ inhibited the growth of U87 in a dose dependent manner. The combine administration of NA-2 (0.33 mM) and temozolomide (0.1 mM) significantly enhanced the cell growth inhibition and apoptosis. Furthermore RT-PCR and imunocytochemistry data revealed that cooperative apoptosis induction was associated with increased ratio of Bax to Bcl-2 and active Caspase-3 expression.

**Conclusion:**

Our findings support that NA-2 possesses strong apoptotic activity and the combined administration of NA-2 and TMZ may be therapeutically exploited for the management of GBM.

**Electronic supplementary material:**

The online version of this article (doi:10.1186/s12935-014-0133-5) contains supplementary material, which is available to authorized users.

## Background

Glioblastoma multiforme (GBM) is a malignant, invasive and most commonly occurring tumor of the central nervous system [1,2]. It accounts for approximately 60% of all malignant primary brain tumors in adults [2]. According to WHO classification of tumors, GBM has been designated as grade IV tumor [3]. GBM has shown poor response to even very aggressive treatment and patients usually have a median survival of approximately 12 to 15 months after diagnosis [4,5]. Current options available for the treatment of GBM (gross total resection along with radio and chemotherapy) are only soothing [4,5]. Although chemotherapeutic agent temozolomide (TMZ) (an oral alkylating agent) has shown some efficacy in delaying the progression of the disease and quality of life, long-lasting responses have not been reported and ultimately patients die of the disease [6]. There are diverse mechanisms of action through which TMZ exerts its anti-tumor effect. TMZ is capable of significantly increasing the sensitivity of O^6^ methyl guanine– DNA methyl transferase (MGMT)-negative GBMs to radiotherapy [7]. This effect of TMZ is produced by its ability to increase the extent of radiation induced double strand DNA damage [7]. To some extent TMZ exerts its cytotoxic activity by pro-autophagic [8] and/or apoptotic pathway [9].

In addition to alkylating agents the use of non-steroidal anti-inflammatory drugs (NSAIDs) and Bevacizumab (BVZ), a humanized monoclonal antibody has also been reported [10-12]. However, the BVZ does not improve survival of patients with newly diagnosed GBM [13] and also demonstrated several side effects including GIT perforation, wound dehiscence, leukoencephalopathy syndrome, intracranial hemorrhage, kidney damage, and heart failure [12,14]. As far as NSAIDs are concerned, although they can alter cell cycle distribution, inhibit cyclins, modulate Bcl-2 family proteins and induce apoptosis [15], their prolong use have been found to be associated with various side effects [16]. Therefore there is a need of developing new compounds which can be use as a single agent therapy or else can be used in combination with low doses of conventional drugs.

N-(2-hydroxyphenyl)acetamide (NA-2) also known as, O-acetaminophenol and 2-acetylaminophenol, is a derivative of salicylic acid which has been reported by Saeed and Saeed [17] as less toxic compound compared to paracetamol or aspirin with an anti-platelet aggregating and anti-inflammatory activity. Apart from this patent application documents, to our knowledge not much work has been done on this compound. This compound was also studied in our laboratory in chronic inflammatory model of pain and it was also observed to show inhibitory affects on inflammatory cytokines and ROS/RNS [18,19]. Therefore in the present study we aimed to explore the activity of NA-2 on growth inhibition of GBM cells when given as a single agent or in combination with TMZ and to examine whether apoptosis is involve in the cell growth inhibition. In our preliminary study NA-2, has shown promising results in apoptotic assay and also exhibited anti-proliferative activity.

## Results

### Growth Inhibitory effect of NA-2 and TMZ alone on U87 GBM cells

**Figure 1:**
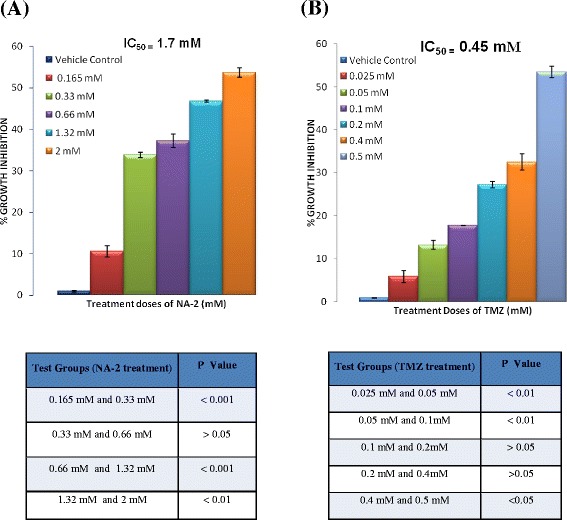
**Effect of NA-2 (A) and TMZ (B) on U87 cells growth inhibition.** Cells were treated with different concentrations of NA-2 and TMZ for 24 hrs and MTT assay was performed. Both NA-2 and TMZ inhibited the growth of U87 cells in concentration dependent manner. Each bar represents mean ± S.E.M of three independent experiments. Significant difference between vehicle control and treated cells are indicated by ***P <0.001.

Following the 24 hrs treatment of U87 GBM cells with varying concentrations of NA-2 and TMZ, the growth inhibitory effect was evaluated using MTT assay. Both NA-2 and TMZ significantly inhibited the growth of U87 cells in dose dependent manner (Figure 1 A & B). ANOVA with bonferroni’s post hoc test was used to assess the level of significance within the test doses and the data is represented in Figure 1.

The cytotoxicity or growth inhibitory activity of NA-2 was also checked on the pancreatic cancer cells (PSN-1 cell line) using the cell titer blue (CTB) assay. Fluorescence was measured on SpectraMax® spectrofluorimeter at 544 nm as an excitation wavelength and 590 nm as an emission wavelength. All measurements were performed in triplicate. The PSN-1 cells were treated with various concentrations of NA-2 (0.165, 0.33, 0.66, 1.32 mM) for 24 hr. A significant concentration-dependent inhibition on the viability of PSN-1 cells was observed (data provided as a Additional file 1: Figure S1).

### Synergistic activity of NA-2 and TMZ

**Figure 2:**
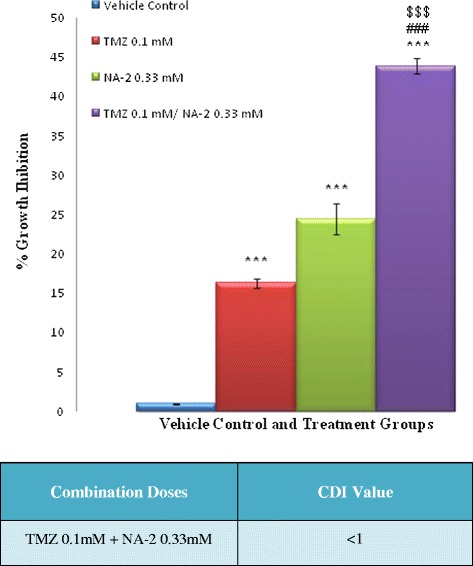
**Effect of NA-2 and TMZ alone and in combinations on U87 cells growth inhibition.** Cells were treated NA-2, TMZ and their combinations for 24 hrs and MTT assay was performed. Significant increase in growth inhibition was observed when TMZ (0.1 mM) combine with 0.33 mM of NA-2. Each bar represents mean ± S.E.M of three independent experiments. One-way ANOVA reveals a significant difference between individual drug treated cells and the cells receiving combined treatment of TMZ/NA-2 (^###^ *P* <0.001). Whereas significant difference between control and treatment groups is indicated by ***P <0.001. Row under the figure represents the coefficient of drug interaction (CDI) = AB/(A × B). According to the absorbance of each group, AB is the ratio of the combination groups to control group; A or B is the ratio of the single agent groups to control group. Therefore, CDI value greater than, equal to or less than 1 specifies that the drugs are antagonistic, additive or synergistic respectively. The results show that TMZ and NA-2 synergistically inhibit growth of U87 cells when given in combination of 0.1 mM and 0.33 mM respectively.

In order to determine whether the growth inhibitory activity of TMZ is enhanced by NA-2, we treated U87 cells with various combinations of these drugs for 24 hrs and MTT assay was performed. Data was analyzed and it was found that growth inhibition increased significantly (P <0.001) when TMZ 0.1 mM combined with 0.33 mM of NA-2, as compared to their individual treatment (Figure 2). Coefficient of drug interaction (CDI) values were also calculated to determine whether the combined effect of TMZ and NA-2 was synergistic, additive or antagonistic. The combine effect of TMZ 0.1 mM with 0.33 mM of NA-2 was found to be synergistic with a CDI value of 0.89. Doses that produce synergistic effect were used for further studies.

### Combined pro-apoptotic effect of NA-2 and TMZ

**Figure 3:**
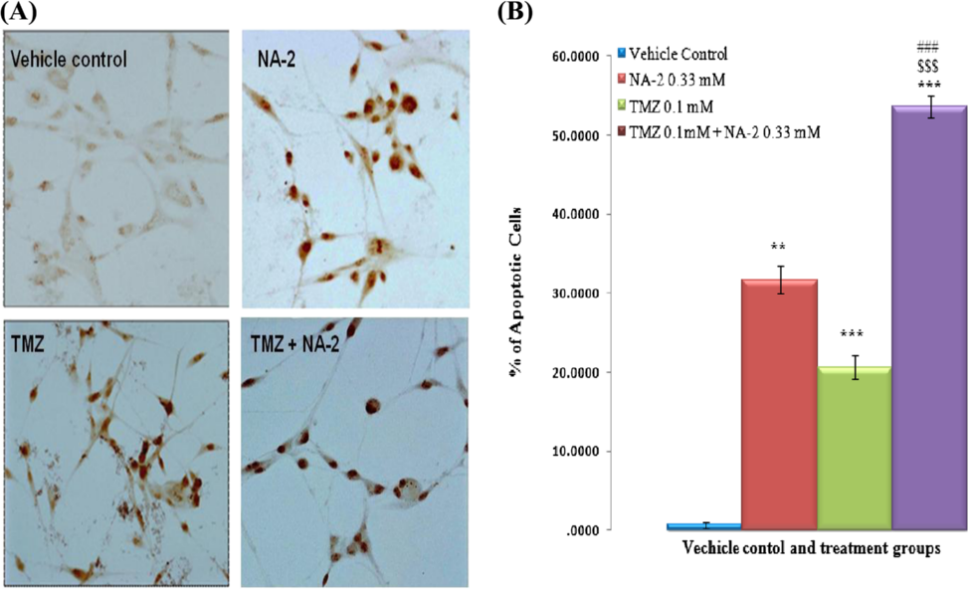
**(A) Representative photomicrographs presenting increased apoptosis in cells exposed to temozolomide in combination with NA-2 revealed by TUNEL assay.** U87 cells were treated with TMZ and NA-2 as single agent or in combination as indicated. After 24 hrs TUNEL method was used to detect apoptotic cells and photographs were taken under the microscope. Note that the dark brown stain is indicative of apoptosis and is strongest in cells treated with TMZ and NA-2 in combination. **(B)** Bar graph showing percentage of apoptotic cells by TUNEL assay and each bar represents mean ± S.E.M of three independent experiments. Significant difference between vehicle control and treated cells is indicated by **P <0.01 and ***P <0.001. Whereas the significant difference between the individual drugs NA-2 or TMZ and their combination treatment is indicated by ^$$$^ *P* <0.001, ^###^ *P* <0.001 respectively.

U87 cells were treated with NA-2 (0.33 mM) and (TMZ 0.1 mM) alone and in combination for 24 hrs and apoptosis was detected by TUNEL assay. We found that combination treatment was more effective to induce apoptosis than the individual treatment of the drug (Figure 3A). Only 31.7% ±1.81% and 20.5% ±1.46% of apoptosis was observed when cells were treated with NA-2 and TMZ alone. Whereas, the percentage of apoptotic cells was dramatically increased to 53.6% ±1.40% in case of combination treatment (Figure 3B).

### Effect of NA-2, TMZ and their combination treatment on Bcl-2, Bax and Caspase-3 expression

A balance between the expression of pro-apoptotic protein (Bax) and anti-apoptotic protein (Bcl-2) plays an important role in the initiation of apoptosis. Caspase 3 being an ultimate executioner is very important for apoptosis. We therefore studied the ratio of Bax to Bcl-2 and caspase-3 gene expressions at transcriptional level in drug treated and control cells by RT-PCR and the level of Bax, Bcl-2 and caspase-3 gene expression was normalized to β-actin.

**Figure 4:**
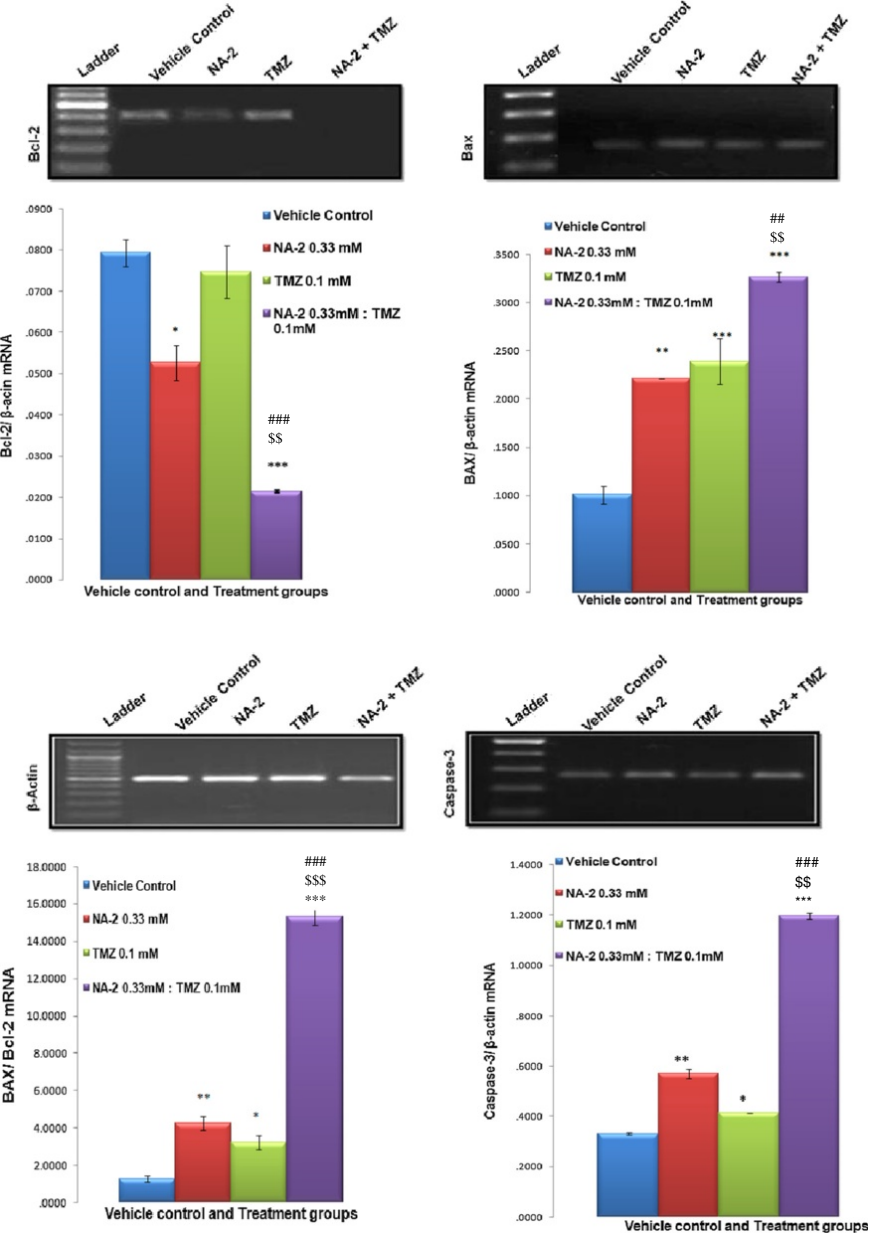
**Effect of NA-2, TMZ and their combination on Bcl-2, Bax and Caspase-3 expression.** Representative bands for BCL-2, BAX and Caspase-3 detected by RT PCR in vehicle control and treated cells. The housekeeping gene β-actin was amplified as an internal control. Graphic illustration of Bcl-2, Bax, Bax/ Bcl-2 ratio and Caspase-3 gene after normalizing with their β-actin expression also shown. Combination treatment with NA-2 and TMZ induce marked changes in the expression of all three mentioned genes as compared to their individual treatment. Each bar represents mean ± S.E.M of three independent experiments. Significant difference between vehicle control and treated cells was indicated by *P <0.05, **P <0.01, ***P <0.001. The significant difference between the individual drugs NA-2 and TMZ and their combination treatment is indicated by ^$$^ *P* <0.01, ^$$$^ *P* <0.001 and ^###^ *P* <0.001 respectively.

Our results clearly shows that treatment with NA-2 alone leads to significant decrease in Bcl-2 expression as compare to control While no significant difference was observed in case of TMZ treatment (Figure 4). Moreover, further decrease in Bcl-2 was observed in the group receiving combination treatment of TMZ with NA-2. On the contrary, combination treatment showed more significant increase in Bax expression as compared to the control and individual drug treatment and these changes in Bcl-2 and Bax gene expression leads to dramatic increase in Bax to Bcl-2 ratio in cells exposed to combined treatment of TMZ and NA-2 than their individual treatment. Likewise, profound increase in caspase-3 expression was observed in cells treated with TMZ and NA-2 in combination.

### Effect of NA-2, TMZ and their combination treatment on active Caspase-3 protein expression

**Figure 5:**
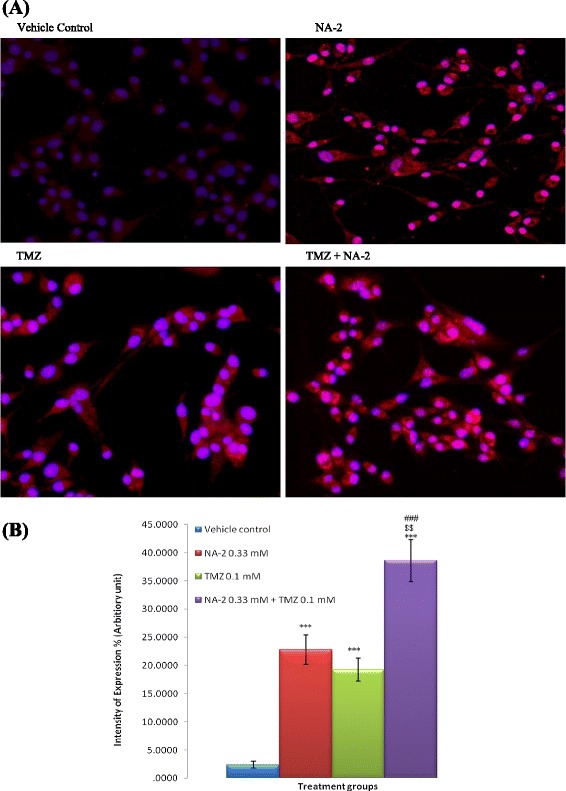
**Effect of NA-2 and TMZ alone and in combination on active caspase-3 expression. (A)** Representative Photomicrographs of cleaved caspase-3 immunoreactivity in control (DMSO treated) and treated group of U87 cells. Base line immunofluorescence was detected in control group. Significant Increase in immunofluorescence is detected in all treated groups with the highest observed intensity in combination treatment group (NA-2 + TMZ). **(B)** Quantification of cleaved caspase-3 expression using Image J software, cells were counted in the field then percent intensity (arbitrary unit) was calculated. Each bar represents mean ± S.E.M of three independent experiments. Significant difference between vehicle control and treated cells was indicated ***P <0.001. The significant difference between the individual drugs NA-2 or TMZ and their combination treatment is indicated by ^$$^ *P* <0.01, and ^###^ *P* <0.001 respectively.

To determine whether caspase-3 play a role in TMZ and/or NA-2 mediated apoptosis of U87 cells, we assessed activated caspase-3 protein level in both control and drug treated groups (NA-2, TMZ and NA-2 + TMZ) using imunocytochemistry. As shown in Figure 5 (A) and (B) treatment of U87 cells with NA-2 and TMZ as single agent leads to minor but significant increase in active caspase-3 protein expression as compare to control. Moreover, marked elevated expression was observed in the group receiving combination treatment of TMZ with NA-2 as compare to control and their individual treatment group.

## Discussion

Although an alkylating agent TMZ has improved the treatment of malignant gliomas, long-term therapeutic responses are not observed and the majority of patients’ die due to aggressive progression of the disease [6]. It is therefore interesting to search for ways to facilitate therapeutic compound and their combination effect with different drugs. In the present study, our results demonstrate that NA-2 and TMZ inhibits the growth of U87 cells in dose dependent manner. The growth inhibitory activity of NA-2 was found to be related to apoptosis. It was also observed that NA-2 possesses synergistic apoptotic activity in combination with TMZ. Our results are supported by the studies demonstrating in vitro apoptosis-inducing ability of NSAIDs [20,21]. Non-steroidal anti-inflammatory drugs such as acetylsalicylic acid (aspirin) has been reported to induce apoptosis in various cancer cell line and known to reduce the viability of Glioblastoma cells to 46% when given at a concentration of 5 mM [22] which is approximately 3 fold higher then the IC50 1.7 mM of NA-2. It induces apoptosis at a dose of 8 mM [22] where as in case of NA-2 only 0.33 mM was sufficient to induce apoptosis in 37.1% cells and increased to 53.6% when given in combination with TMZ.

Apoptosis or programmed cell death is an important physiological process that has a crucial role in development and tissue homeostasis [23]. Nevertheless, it is also involved in various pathological conditions. Its defects are common occurrence in oncogenesis and contribute to drug resistance [24]. It is also found to be deregulated in gliomas [25] and therefore it is interesting to target underlying molecular markers involved in the apoptotic process. Class of cysteine protease called caspases and Bcl-2 family proteins [25] are important group of proteins involved in apoptotic cell death [26]. Pro-apoptotic (Bax) and anti-apoptotic (Bcl-2) proteins are subgroup of Bcl-2 family proteins that promotes and inhibit apoptosis respectively [27]. Increased levels of Bcl-2 protein is observed in many tumor cells, contributes to increased tumor cell resistance and tumor growth by decreasing Bax-Bcl-2 ratio. Bax in itself does not cause cell death, but its elevated expression favors an entry into the apoptotic program following a death signal by countering Bcl-2 activity. Thus, tilting the intracellular balance towards an increased Bax-Bcl-2 ratio which can occur through either elevated synthesis of Bax or through decreased synthesis of Bcl-2 will favor apoptosis and thus reduce the rate of survival [28]. Keeping these in mind, the present study was designed to study the expression pattern of Bax, and Bcl-2 following the treatments given to U87 cell lines. Current findings of our experiments demonstrated that NA-2 up-regulated Bax and down regulated Bcl-2 expression at transcriptional level. Whereas TMZ has shown to increase Bax expression only and has shown no significant effect on Bcl-2 expression. The effect on the expression of these markers was more pronounced in cells treated with combination of NA-2 and TMZ. The up-regulated Bax expression by NA-2 and TMZ and down-regulated Bcl-2 expression by NA-2 only leads to dramatic increase in Bax-Bcl-2 ratio as compared to control and thus apoptosis. Our results are supported by other studies which shows that NSAIDs like selective COX-2 inhibitors NS-398 and SC-58125 can down regulate Bcl-2 and subsequently induce apoptosis in colon and prostrate cancer cell lines [29]. Whereas celecoxib, indomethacin and aspirin could induce apoptosis by up-regulating Bax and activation of caspase-3 [30,31]. Mahd et al. [32] and Hossain et al. [33] has shown that calcium salicylate and aspirin increase Bax to Bcl-2 ratio in fibrosarcoma and hepatocellular carcinoma cells by down and up regulating Bcl-2 and Bax respectively. In case of glioblastoma cells 8 mM of asprin reduced Bcl-2 expression to 42% after 48 hrs whereas only 0.33 mM NA-2 inhibited Bcl-2 expression to approximately 35% as compare to control which was increased to 96% when given in combination with TMZ. Anti-apoptotic Bcl-2 protein has been found to prevent both the loss of the mitochondrial membrane potential and the efflux of cytochrome c release and inhibit apoptosis. Bax antagonizes the effect of Bcl-2 that results in release of cytochrome c in cytosol. Once cytochrome c is released in the cytosol it contributes to the formation of apoptosome and subsequent activation of caspases [34].

Caspases, especially caspase-3, are known to act downstream of Bax/Bcl-2 control and play a key role in the execution of apoptosis; responsible for proteolytic cleavage of many proteins [26]. Therefore we also studied the expression of active caspase-3. An enhanced expression of active caspase-3 was observed after treatment with NA-2 and TMZ and the effect was further enhanced when cells were treated in combination. This increased expression of activated caspase-3 in treated groups as compare to controls may be a consequence of increased Bax to Bcl-2 ratio as explained above and also the highest activated caspase-3 expression was observed in a combination treatment group where Bax-bcl-2 ratio was highest. Furthermore several studies have shown NSAIDs like celecoxib, asprin, indomethacin and nitric oxide giving-NSAID can increase activation and expression of Caspase-3 [10,35]. Nevertheless, further studies are needed to completely understand the mechanism of action of NA-2 and its synergy with TMZ.

## Conclusion

These results clearly reflect apoptotic activities of NA-2. It also demonstrates that both these activities of NA-2 can be refined further when given in combination with TMZ. This may in part be related to the increase in Bax/Bcl-2 ratio and active Caspase-3 expression.

In future we plan to test the efficacy and mechanism of action of NA-2 on other cell lines of the similar tumor types as well as on various other tumor cells to check if the activity of the NA-2 is tumor specific or it also inhibit the other tumor types. Further we also want to explore its action in in-vivo model of glioblastoma multiforme.

## Methods

### Drug/compound preparation

Test drug N-(2-hydroxyphenyl)acetamide and temozolomide were purchased from Sigma Chemical Company (St. Louis, MO, USA). Stock concentrations of NA-2 (2000 mM) and TMZ (128 mM) were prepared in sterile 100% DMSO and stored at −20°C. The working solutions of the drugs were prepared fresh from stock solutions by diluting the stock in Dulbecco modified Eagle medium (DMEM). Six different working concentrations of NA-2 (0.165 mM, 0.33 mM, 0.66 mM, 1.32 mM, 2 mM) and TMZ (0.025 mM, 0.05 mM, 0.1 mM, 0.2 mM, 0.4 mM, 0.5 mM) were used in the study. In addition to the above mentioned concentrations, cells were also treated with various combinations of NA-2 and TMZ (0.33 mM +0.1 mM, 0.33 mM +0.2 mM, 0.66 mM +0.1 mM, 0.66 mM +0.2 mM, 1.32 mM +0.1 mM and 1.32 mM +0.2 mM) in order to evaluate their combined effect. Cells treated with only DMSO (0.1%) were used as controls.

### Cell cultures

Human glioblastoma cell line U87 (ATCC number: HTB-14™) was purchased from American Type Tissue Culture Collection (ATCC, USA) and cultured in DMEM (Sigma Chemicals) supplemented with 1% penicillin and streptomycin, 1%, amphotericin B, 1% sodium pyruvate, 1% L-glutamine and 10% fetal bovine serum FBS in a humidified atmosphere at 37°C containing 5% CO_2_.

### (4, 5-dimethyl thiazol-2-yl)-2, 5-diphenyl tetrazolium bromide (MTT) Assay

The effect of NA-2 and TMZ as a single agent and in various combinations (mentioned above) on GBM tumor cell growth was evaluated by the MTT assay [36]. Briefly, monolayer of the cells was trypsinized and re-suspended in medium containing 10% FBS. The cells (3 × 10^3^ cells/100 μl) were plated in 96-wells plate and incubated in humidified atmosphere at 37°C containing 5% CO_2_ for 24 hrs. Following incubation, media was aspirated and 100 μl of different test concentrations of NA-2 and TMZ and their various combinations (mentioned above) in media containing 1% FBS were added. Maximum DMSO concentration used was less than 0.1%. Cells treated with 0.1% DMSO in media (1% FBS) were used as untreated controls. The plates were re-incubated for 24 hrs and after incubation, sample solution in the wells was flicked off and 100 μl of 0.5 mg/ml MTT dye (Promega, USA) was added to each well and further incubated for 3 hrs at 37°C in 5% CO_2_. After incubation, supernatant was removed and 100 μl of DMSO (100%) was added to each well to solubilize formazan. The absorbance was recorded on 96-wells plate ELISA reader at 490 nm [37]. The percentage of viable cells following treatment was normalized to untreated controls. All assays were performed in tetraplicates and the percentage growth inhibition was calculated using the formula [38]:

$$ \%\;\mathrm{c}\mathrm{ell}\;\mathrm{inhibition} = 100\hbox{-} \left\{\left(\mathrm{A}\mathrm{t}\hbox{-} \mathrm{Ab}\right)/\left(\mathrm{A}\mathrm{c}\;\hbox{-}\;\mathrm{Ab}\right)\right\}\times 100 $$

Where, At, Ab, and Ac stands for absorbance value of test compound (cells + media + drug), absorbance value of blank (media + drug), and absorbance value of control (cells + media + vehicle) respectively.

### Synergy analysis of combine drug effects

The coefficient of drug interaction (CDI) was used to determine the synergistically inhibitory effect of drug combination, which was calculated as [39]; CDI = AB/(A × B)

Where, AB, and A or B designate the ratio of the combination groups to control group (DMSO) and the ratio of single agent (drug) to control group (DMSO) respectively. Thus a CDI value less than, equal to or greater than 1 specifies that the drugs are synergistic, additive or antagonistic, respectively [40].

### Detection of apoptosis by terminal deoxynucleotidyl transferase dUTP nick end labeling (TUNEL) assay

U87 glioblastoma cells were grown in two chambered slides (Lab-Tek®) and treated with NA-2 (0.33 mM) and TMZ (0.1 mM) alone, and in combination (NA-2 0.33 mM + TMZ 0.1 mM) and DMSO for 24 hrs at 37°C and 5% CO_2_. After incubation, DeadEnd™ TUNEL assay system kit (Promega Corporation, USA) was used to detect apoptosis in cell as per manufacturer protocol. Cells with dark brown nuclei were considered apoptotic [41]. Quantification of apoptotic cells was done by counting total cells and apoptotic cells in five blindly selected fields scoring between 350 and 550 cells, and the number of apoptotic cells was expressed as percentages of the total cell population [42]. Data from three different experiments were combined and expressed as means ± SEM.

### Semi-quantitative RT-PCR

Cells (1×10^6^) were treated for 24 hrs with drugs alone and their effective combination. Cells treated with DMSO only served as untreated control. After 24 hours media was aspirated, cells were trypsinized and centrifuged to get the cell pellets. The RNA was then isolated using SV Total RNA Isolation System (Promega, USA) kit according to the manufacturer׳' protocol. The concentration and purity of isolated RNA was assessed using spectrophotometer (UV-Spec-1700, Shimadzu). The isolated RNA samples (500 ng) were reverse-transcribed into cDNA using RevertAid™ First Strand cDNA Synthesis Kit (Fermentas, Maryland, USA). Negative control was also run including RT reaction mixture omitting reverse transcriptase. The transcribed cDNA (1 μl) was then amplified using PCR Master Mix (Fermentas, USA) and oligonucleotide primers corresponding to transcripts of the respective genes. The PCR reaction products were electrophoretically resolved on ethidium bromide stained 1% agarose gel and densitometric analysis was performed using Quantichrome software. Density of each mRNA band was normalized to its respective housekeeping gene i.e. β-actin. The primer sequences used in this study were Bax (sense, 5’-AAGAAGCTGAGCGAGTGTC-3’) (antisense, 5’-GGCCCCAGTTGAAGTTGC-3’), Bcl-2 (sense, 5’-ACTTCGCCGAGATGTCCAGC-3’) (antisense, 5’-GGCAGGCATGTTGACTTCAC-3’), Caspase-3 (sense, 5’-TTCAGAGGGGATCGTTGTAGAAGTC-3’) (antisense, 5’- CAAGCTTGTCGGCATACTGTTTCAG-3’), β-Actin (sense, 5’-GTCCTGTGGCATCCACGAAAC-3’) (antisense, 5’ -GCTCCAACCGACTGCTGTCA-3’) [43-45].

### Immunocytochemical analysis

For imunocytochemistry, cells were plated in two chambered slides (Lab-Tek®) and incubated with NA-2 (0.33 mM), TMZ (0.1 mM) alone, their combination (NA-2 0.33 mM + TMZ 0.1 mM) and DMSO (untreated control) at 37°C and 5% CO_2_. After 24 hrs cells were fixed with 4% paraformaldehyde, washed five times with PBS, blocked with blocking solution (2% bovine serum albumin (BSA),2% normal goat serum and 0.2%Tween20 all prepared in PBS) at 37°C for 1 hr. Next, cells were washed with PBS five times and incubated with rabbit anti-cleaved caspase-3 (Santa Cruz Biotechnology) in 1:100 dilutions at 4°C overnight. On subsequent day cells were washed with PBS three times and then incubate for 1 hr at room temperature with secondary antibody Alexa Flour®^546^ anti-rabbit IgG at 1:200 dilution in PBS. Then cells were washed again and counterstained with 4, 6-diamidino-2-phenylindole (DAPI) in 1:500 dilutions in PBS). The stained slides were then mounted with PBS/glycerol (1:1) and viewed under a fluorescent microscope (NIKON).

For quantification studies, the images were analyzed using ImageJ software (National Institutes of Health, USA) [46]. Briefly, 5 fields were selected blind based and the intensity of fluorescence was quantified in cells and subsequently the background intensity was subtracted from it, the remaining particles were considered to represent cleaved caspase-3. Cells were counted and percentage intensity of expression was calculated and means percentage intensity was found out. Data from three different experiments were combined and expressed as means ± SEM.

### Statistical analysis

The results obtained from various experiments were analyzed with SPSS-19 software. Data were expressed as mean ± standard error of the mean (SEM) of separate experiments (n ≥3) and compared by one-way analysis of variance (ANOVA) followed by Bonferoni post hoc test. P <0.05 was considered to be statistically significant.

## Additional file

Additional file 1: Figure S1. Effect of NA-2 on PSN-1 cells growth inhibition. Cells were treated with different concentrations of NA-2 for 24 hrs and cell titer blue (CTB) assay was performed. All measurements were performed in triplicate. NA-2 inhibited the growth of PSN-1 cells in concentration dependent manner. A significant concentration-dependent inhibition on the viability of PSN-1 cells was observed. Each bar represents mean ± S.E.M of three independent experiments.

## Abbreviations

GBM: Glioblastoma multiforme
TMZ: Temozolomide
MGMT: O^6^ methyl guanine– DNA methyl transferase
NSAIDs: Non-steroidal anti-inflammatory drugs
NA-2: N-(2-hydroxyphenyl) acetamide
MTT: (4, 5-dimethyl thiazol-2-yl)-2, 5-diphenyl tetrazolium bromide
TUNEL: Terminal deoxynucleotidyl transferase dUTP nick end labeling
CDI: Coefficient of drug interaction
Bcl-2: B-cell lymphoma 2
Bax: B-cell lymphoma associated X
