# Supplementary material for: N-(2-hydroxyphenyl)acetamide (NA-2) and Temozolomide synergistically induce apoptosis in human glioblastoma cell line U87
Source: Cancer Cell Int. 2014 Nov 30;14:133. doi: 10.1186/s12935-014-0133-5 (PMC4319240; doi:10.1186/s12935-014-0133-5)
Supplement: Additional file 1: Figure S1. — Effect of NA-2 on PSN-1 cells growth inhibition. Cells were treated with different concentrations of NA-2 for 24 hrs and cell titer blue (CTB) assay was performed. All measurements were performed in triplicate. NA-2 inhibited the growth of PSN-1 cells in concentration dependent manner. A significant concentration-dependent inhibition on the viability of PSN-1 cells was observed. Each bar represents mean ± S.E.M of three independent experiments. [file 12935_2014_133_MOESM1_ESM.docx]

**Additional file 1 Figure: Effect of NA-2 on PSN-1 cells growth inhibition**. Cells were treated with different concentrations of NA-2 for 24 hrs and cell titer blue (CTB) assay was performed. All measurements were performed in triplicate. NA-2 inhibited the growth of PSN-1 cells in concentration dependent manner. A significant concentration-dependent inhibition on the viability of PSN-1 cells was observed. Each bar represents mean ± S.E.M of three independent experiments.
